# Alcohol in Fever

**Published:** 1879-11-20

**Authors:** 


### ALCOHOL IN FEVER.

In a discussion, at a recent meeting of the British Medical Association, Dr. Spedding (Belfast) said he had considerable experience in the administration of stimulants to children in a variety of diseases, chiefly high pyrexiæ and fevers. Only one speaker had alluded to giving stimulants to children, and he seemed to be rather averse to it. He had cases in which, for several days, children had not allowed one particle of nutritive food to enter their stomachs, and had lived on nothing but water and punch, made very sweet and taken cold. It had been his practice for the last eight years, in the Belfast Dispensary, to withhold alcohol till pulmonary complications appeared, about the second or third week. He thought that was the time when the use of stimulants was indicated; and then he measured the amount of his stimulants by the amount of the pulmonary complications. It seemed to act remarkably well on the heart, and also acted as an expectorant.

Dr. Smith, of Dumfries, said that in Scotland they recognized fever as a disease that ran a certain course. They found that the best method of preventing death was to support the system from the commencement of the fever; and they did not give stimulants when they found that nervous symptoms were produced.

Dr. Sinclair (Dundee) was of opinion that the other ingredients of ignored, as they too often were. Whisky contained none of those volatile ethers which were developed by keeping in the rich wines of France and Spain; and for this reason he objected to the administra- most alcoholic fluids were not by any means to be under-estimated or tion of whisky.

Dr. Little (Dublin) wished it not to be supposed that he thought alcohol was not one of the most valuable agents they had. He had, however, thought it was potent for evil as well as for good; and that they must be exceedingly cautious in its use. He had himself been in the habit of prescribing a cup of tea in the morning, beaten up with the yolk of an egg, as a substitute for stimulants.

The President said this had been the most satisfactory discussion on a question of treatment that he had ever heard. They had gentlemen there with extreme views on both sides; and there had arisen from those gentlemen not only a convergence, but an actual conjunction of opinion as to the point of the value of alcohol in the treatment of fever. In the first place, it was apparently agreed that the patient in fever was like a ship in the storm. They could not do very much for the storm, but they could do a great deal in steering the ship in the storm. The main object was to support the life of the patient in passing through the storm of fever. In the next place, when the patient began to sail, and death was threatened, perhaps the best remedy was alcohol. It was also agreed that circumstances arose in the course of the fever, and pointing to failure, perhaps not only was alcohol a successful, but was the only successful means which they could employ; and that, when they gave alcohol, they must give it with a sparing or with a tentative hand; and so long as certain evidences of disagreeament did not arise; so long as the tongue was not dry, as the pulse was not increased; so long as no suppression of urine, and as no increase of delirium occurred; and so long as the patient felt comforted and quiet, they might go on with the idea that they were assisting the patient in arriving at a happy termination of the disease.

Dr. Morell Mackenzie read an article on Laryngeal Phthisis, of which the following is an abstract:

1. Laryngeal phthisis is due to the presence and subsequent breaking down of tubercles in the mucous and sub-mucous membranes. The tubercles, some very small and some as large as a millet seed, are found imbedded in a reticular structure, filled with small, round lymphoid cells. This tubercular matter is sometimes deposited uniformly through the thickness of the mucous membrane, but much more commonly it is found in the most superficial layer of the mucous membrane, immediately beneath the epithelium. 2. Laryngeal phthisis is essentially a secondary phenomenon, occurring as a sequel to pulmonary phthisis. There is no evidence that any case of primary laryngeal phthisis has ever existed. 3. The disease is not due to the corrosive action of the sputa. 4. The disease is much more common among males than females. Out of 500 cases examined by the author during life, 365 were males and 135 females. In a hundred necropsies, there were 73 males and 23 females. 5. The most frequently present symptom of Laryngeal phthisis is impairment of the local function. In 500 cases the voice was impaired 460 times; cough was a marked symptom in 427 patients; dysphagia occurred 151 times. 6. The naked eye appearances of Laryngeal phthisis, either during life or after death, cannot be absolutely relied upon, but pale pyriform swellings of the aryepiglottic folds, and a pale, turban-like thickening of the epiglottis, are seldom met with except in Laryngeal phthisis. More or less uniform thickening, with marked pallor, of the mucous membrane and small scattered ulcers, are the characteristic features of the disease. 7. The prognosis is always unfavorable, the ordinary duration of life after the throat symptoms have become troublesome being from twelve to eighteen months. 8. The only treatment which is of any use consists in the employment of palliative remedies. Where there is a pain in swallowing, insufflation of morphia gives the greatest amount of relief.

